# Building sustainability indicators in the health dimension for solid waste management**^1^**

**DOI:** 10.1590/1518-8345.0635.2732

**Published:** 2016-08-08

**Authors:** Tatiane Bonametti Veiga, Silvano da Silva Coutinho, Silvia Carla Silva Andre, Adriana Aparecida Mendes, Angela Maria Magosso Takayanagui

**Affiliations:** 2 PhD, Professor Adjunto, Departamento de Engenharia Ambiental, Universidade Estadual do Centro-Oeste, Irati, PR, Brazil.; 3 PhD, Adjunct Professor, Departamento de Educação Física, Universidade Estadual do Centro-Oeste, Irati, PR, Brazil.; 4 PhD, Adjunct Professor, Departamento de Enfermagem, Universidade Federal de São Carlos, São Carlos, SP, Brazil.; 5 PhD, Adjunct Professor, Departamento de Enfermagem, Centro Universitário de Araraquara, Araraquara, SP, Brazil.; 6 PhD, Associate Retired Professor, Escola de Enfermagem de Ribeirão Preto, Universidade de São Paulo, Centro Colaborador da OPAS/OMS para o Desenvolvimento da Pesquisa em Enfermagem, Ribeirão Preto, SP, Brazil.

**Keywords:** Sustainable Development, Sustainable Development Indicators, Solid Waste, Environmental Health, Public Health

## Abstract

**Objective:**

to prepare a list of sustainability indicators in the health dimension, for urban solid waste management.

**Methods:**

a descriptive and exploratory study performed jointly with 52 solid waste specialists, using a three-steps Delphi technique, and a scale measuring the degree of importance for agreement among the researchers in this area.

**Results:**

the subjects under study were 92,3% PhD's concentrated in the age group from 30 to 40 years old (32,7%) and 51% were men. At the end of the 3rd step of the Delphi process, the average and standard deviation of all the proposed indicators varied from 4,22 (±0,79) to 4,72 (±0,64), in a scale of scores for each indicator from 1 to 5 (from "dispensable" to "very important"). Results showed the level of correspondence among the participants ranging from 82% to 94% related to those indicators.

**Conclusion:**

the proposed indicators may be helpful not only for the identification of data that is updated in this area, but also to enlarge the field of debates of the environmental health policies, directed not only for urban solid waste but for the achievement of better health conditions for the Brazilian context.

## Introduction

The needs that derive from human development, change the environment and alter substantially the natural conditions. In the face of these shifts, there is a need of new and permanent investments to improve the sanitation conditions. The sanitation movement foster changes in the mindset about health and illness^1^ and may help to minimize the rate of those illnesses linked to inadequate sewage.^2^ ^-^ ^3^ as these shortcomings in infrastructure impact in the health conditions of the populations^4^ ^-^ ^5^.

Among the different areas of sanitation activities, waste management is considered a relevant and present problem for society as a whole, as it is an essential service for public health in developing countries^6^. This area of concern is a constant in the political and administrative agendas in several countries. In Brazil, the debated around waste management were the origin of many studies in the last years^7^ ^-^ ^8^ and were the basis for the National Policy for Solid Waste that was enacted by Law 12.305 in August 2^nd^ 2010^9^.

This policy was of great importance for the improvement of concept standardization in waste management, providing criteria about adequate and safe management and an integrated and sustainable administration. Additionally the policy mandates every state and municipal authority to establish a plan for integrated urban solid waste management, to optimize this management, in order to provoke several beneficial effects for the whole Brazilian society^10^.

The demand for having a waste management plan, proposed by the National Policy for Solid Wasteis also mandatory for the facilities that generate hazardous waste such as health services, that pose public health or environmental risks, because they have one or more characteristics e.g. flammability, corrosiveness, reactivity, toxicity, pathogenicity, carcinogenicity, teratogenicity and mutagenicity^9^. Around 25% of the health service waste is considered hazardous with potential risks to workers and that risk may extend also to the community^11^. This waste is classified in five groups: A - biologic, B - chemical, C - radioactive, D - common and E - sharps, being essential to develop studies to create protocols and new practices to manage these kind of waste^7^.

The National Policy for Solid Wastealso reaffirms the relevance of the rolling out of the management plan in the facilities that generate health services related waste^9^. Through resolutions 306/2004 from Health Surveillance Agency (ANVISA) and 358/2005 from the National Environmental Council (CONAMA), apart from allocating responsibility of management for this waste to the respective generator, also state the different steps for its management, in order to reduce environmental pollution, and to protect the agents that work in all the phases of the process^7^ ^-^ ^8^. 

To cope with the new Brazilian legal requirements, new and permanent investments will be needed in order to have a accurate description of reality. In this context, building indicators is a useful tool to help in gathering a set of updated information to improve the waste management^12^, and minimize possible impacts in health and environment. This information is essential because it will support the managers for planning and interventions in the decision-making process. The sustainability indicators have different approaches, depending on the dimensions where they belong, such as environmental, economics, and social^13^ ^)^ as well as other relevant dimensions as institutional and health dimensions.

## Methods 

This is a descriptive and exploratory research, with the participation of specialists in the area of waste management. Due to the nature of the findings, the quantitative approach was used in the first three stages and then the qualitative one was used for the data collected in the first stage. Is noteworthy that the qualitative and quantitative approaches are not excluding each other^14^ and may be complementary in the quest for better grasp of a certain context, contributing for analyses and proposals for solving problems in health^15^. 

To get to contact the subjects of this research, a query in the directory of Brazilian Research Groups pertaining to the database of the National Council of Scientific and Technological Council (CNPq) was performed, identifying 74 Research Groups with research lines in this area. We sent an invitation to the coordinators of those groups through email, asking them to nominate other new participants from different CNPq research groups, in a snowball strategy^6^, thus expanding the list of invitees to include other subjects that may contribute to the research. 

The inclusion criteria included as a priority, researchers of the Research Groups and experience in activities of waste management, as listed in the database of researchers called *Curriculum Lattes*, following the prescriptions of the technique that presuppose the participation of specialists with theoretical and practical knowledge of the research topics^17^.

Data collection was done through the Delphi technique in three steps with the participation of 52 subjects, followed by the quitting of one in the second step and another one in the third step. When the research was finished 50 subjects remained.

The technique is a methodological strategy for research including a wide group of subjects, that is oriented to frame the judgments of specialists for a maximum of agreement on the studied area, promoting the convergence of opinions^17^. This methodology tries to keep the anonymity to eliminate the influence of factors such as academic or professional status, allowing for a more active and less biased participation, non inhibited as may happen in face-to-face meetings^18^. 

Following the steps of the Delphi technique, participants allocated a specific degree of importance to each indicator, using values from 1 "dispensable" to 5 "very important". In the first step, additionally to this allocation of importance to the five proposed indicators, the participants were asked to suggest the insertion of new indicators, according with a document review done by the researchers, or to propose changes in the texts of the indicators that were presented to them.

One of the key characteristics of the technique is the feedback, that allows the specialists to review the results of the previous step, and to weigh their judgments according to the results of the group^17^. Using this procedure in an iterative process, some points of view were deleted, altered or added following the reflections of the participants. 

The data coming from each step were organized in spreadsheets Microsoft Excel*^(c)^* through double data entry and validation to control for typewriting mistakes. Data were processed through software *Statistical Package for Science Social* (SPSS), ver.19.0. The cutting point was at least 75% of the subjects' evaluation in degrees 4 "important" and 5" very important".

In the first step, the study performed an additional qualitative analysis based in contents examination, to categorize the contributions submitted by the subjects, to avoid duplications of indicators that may affect the length and the friendliness of the instrument, in order to allow a lesser degree of desistance. The ethical aspects were followed according to Resolution 196/96 of the National Council of Health and the proposal of this study was submitted to the Ethics Research Committee of the Nursing School, Ribeirao Preto, University of Sao Paulo, filed under #239, December 14^th^ 2012. Data were collected between March 2013 and February 2014.

## Results

The profile of the 52 interviewees that participated in at least one of the stages of the Delphi technique follows. Among the participants in the research, 51,9% were men, ages in the range from 29 and 70 years old, and a strong participation in age group from 30 to 40 years old (32,7%). Related to the participation in Research Groups, 23,1% were only participants, and 76,79% were coordinators. Following the criteria used, the region with more representatives was the Northeast region (44,2%) (Table 1).

All subjects had at least a Master's Degree, 92,3% had PhD's and 21,2% Post-Doctorate diplomas. Most of them had activities in graduate and post-graduate courses in Federal institutions (55,8%) and 90,4% of these subjects were lecturers in disciplines in the area of waste management.

**Table 1:** Profile of the specialists in waste management participating in the research, Brazil, 2013.

Variables		n	%
Gender (n=52)			
	Feminine	25	48,1
	Masculine	27	51,9
Age groups (n=52)			
	29-40	17	32,7
	41-50	15	28,8
	51-60	14	26,9
	61-70	6	11,6
Activities in Research Groups (n=52)			
	Coordinators	40	76,9
	Researchers	12	23,1
Number of researchers by Region (n=52)			
	North	3	5,7
	Northeast	23	44,2
	Center West	1	1,9
	Southeast	14	27,0
	South	11	21,2
Profession (n=52)			
	Civil Engineer	23	44,2
	Chemical Engineer	4	7,7
	Sanitation Engineer	4	7,7
	Environmental Engineer	3	5,8
	Law Degree	3	5,8
	Other	15	28,8
Masters' area (n=51)			
	Hydraulics and Sanitation	13	25,5
	Civil Engineering	10	19,6
	Environmental Engineering	7	13,7
	Human Development	4	7,9
	Other	17	33,3
PhD area (n=48)			
	Civil Engineering	10	20,8
	Hydraulics and Sanitation	8	16,7
	Public Health	5	10,4
	Geosciences	5	10,4
	Environmental Engineering	5	10,4
	Other	15	31,3
Post-Doc area(n=11)			
	Environmental Engineering	4	36,3
	Hydraulics and Sanitation	3	27,3
	Solid Waste	2	18,2
	Geosciences	1	9,1
	Environmental Engineering	1	9,1

Something worth of note is the variety in the participants' previous trainings, both in graduate and post-graduate levels, allowing for many visions and academic trajectories, thus offering a multidisciplinary contribution in building the indicators. 

In the first step of the Delphi technique, several contributions were collected, and the suggestion of indicators was concurrent among the participants even though there was not communication between them as it happens in the face-to-face techniques. The contents' analysis of 22 suggestions for indicators in the health dimension for solid waste management, lead to the construction of eight new indicators and to change two of the already proposed.

Using the criterion of agreement of at least 75% of the subjects, the indicator "number of working days lost by disease in employees of urban solid waste management (USW) or street cleaning" was excluded in this step, because it did not reach the level as pre-determined in the study (67,3%).

Following this path, the suggestions of the participants in the first step were the basis for building Instrument number 2. In the second step of the technique there was no exclusion of indicators and level of agreement ranged from 76,5% to 96,1%.

All participants received a communication with the results of the second step before completing the third instrument, re-evaluating the proposed surviving indicators that resulted from the previous steps. This feedback allowed the participants to review their opinions related to each indicator, something that is a cornerstone in using the Delphi technique. 

After evaluating the data coming from the second steps, subjects allocated a certain degree of importance to each indicator, ranging from 82% to 94% for degrees 4 and 5. On Table 2 is shown a summary of the results of each step, and the indicators that remained at the end of the Delphi technique. 

**Table 2:** Distribution of degree of importance of the summary of the sustainability indicators in the health dimension for urban solid waste management, as per judgment of specialists in waste management, Brazil, 2013

Indicators		1^st^ Step	2^nd^ Step	3^rd^ Step
Heath Dimension		52 subjects	51 subjects	50 subjects
		% ∑ 4 e 5	% ∑ 4 e 5	% ∑ 4 e 5
1	Number of accidents that involve Informal Refuse Collectors and employees working in USW* management, related to the total number of employees working in these sectors	76,9	88,2	94
2	Number of risk situations happening for Informal Refuse Collectors and employees working in USW management.	84,6	84,3	82
3	Percentage of Informal Refuse Collectors and employees working in USW management, using Personal Protection Equipment.	Included	86,3	84
4	Percentage of Informal Refuse Collectors and employees working in USW management with updated vaccination and check-ups.	Included	78,4	86
5	Number of Dengue foci or proliferation of other types of vectors due to storage and disposal of waste in Informal Refuse Collectors' households or other non-appropriate places.	Included	76,5	90
6	Number of yearly cases of disease related to bad practices of management and disposal of USW.	Included	90,2	84
7	Number of yearly deaths related to related to bad practices of management and disposal of USW.	Included	84,3	82
8	Daily per capita mass of HSSW^†^ related to urban population.	90,4	90,2	92
9	Daily per capita mass of Hazardous Waste (except HSSW) collected, related to urban population.	Included	84,3	90
10	Percentage of HSSW with treatment and environmentally adequate terminal disposal.	Included	96,1	94
11	Percentage of Hazardous Waste (except HSSW) with treatment and environmentally adequate terminal disposal.	Included	94,1	90
12	Percentage of HSSW collected related to the total amount of USW generated.	76,9	76,5	92
13	Number of working days lost by disease in employees of urban solid waste management (USW) or street cleaning.	67,3	Excluded	Excluded

*Urban Solid Waste (USW).

†Health Services Solid Waste (HSSW).

At the end of the study, 12 new indicators were proposed in the health dimension for waste management. Taking into consideration the scale of score for each indicator, the average and the standard deviation of all the indicators in the last step of the technique ranged from 4,22 (±0,79) to 4,72 (±0,64) (Table 3).

**Table 3:** Indicators of sustainability in the health dimension for urban solid waste management as prepared by the Delphi technique following experts' agreement in a concurrence Likert scale from 1 to 5. Brazil, 2013.

Indicators		Average ± SD*
Health Dimension		
1	Number of accidents that involve Informal Refuse Collectors and employees working in USW^†^ management, related to the total number of employees working in these sectors.	4,42±0,73
2	Number of risk situations happening for Informal Refuse Collectors and employees working in USW management.	4,22±0,79
3	Percentage of Informal Refuse Collectors and employees working in USW management, using Personal Protection Equipment.	4,34±0,92
4	Percentage of Informal Refuse Collectors and employees working in USW management with updated vaccination and check-ups.	4,28±0,93
5	Number of Dengue foci or proliferation of other types of vectors due to storage and disposal of waste in Informal Refuse Collectors' households or other non-appropriate places.	4,42±0,84
6	Number of yearly cases of disease related to bad practices of management and disposal of USW.	4,32±0,98
7	Number of yearly deaths related to related to bad practices of management and disposal of USW.	4,34±0,96
8	Daily per capita mass of HSSW^‡^ related to urban population.	4,42±0,84
9	Daily per capita mass of Hazardous Waste (except HSSW) collected, related to urban population.	4,36±1,01
10	Percentage of HSSW with treatment and environmentally adequate terminal disposal.	4,72±0,64
11	Percentage of Hazardous Waste (except HSSW) with treatment and environmentally adequate terminal disposal.	4,56±0,97
12	Percentage of HSSW collected related to the total amount of USW generated.	4,46±0,76

*Standard Deviation.

†Urban Solid Waste (USW).

‡Health Services Sold Waste (HSSW).

## Discussion 

The issue of urban solid waste is considered a public health problem that involves multiple topics of collective interest, being crucial to adopt public policies to support the managers' decisions and the activities of the civil society around waste management^19^, in order to avoid harmful effects to the environment and human health^19^,

In this context, the experts point to the need of larger investments for developing studies to promote building and using sustainability indicators in this particular area^6^ ^,^ ^20^ ^)^ and also concur in relating inadequate sanitation conditions with vector proliferation and expansion of several kind of diseases^2^ ^-^ ^3^. In spite of this, the health dimension in waste management is still poorly discussed and disseminated.

Even though this debate is its early stages, it was possible to verify the interest that the researchers with different backgrounds, put in the process of building the proposed indicators, arriving to higher level of agreement (82% to 94%) for the 12 indicators put to consideration. Another relevant factor is referred to the exclusion of factors, as just one of the indicators was deleted during the process because it did not reach the minimum agreement among the participants, something that underlines the need to incorporate of concepts that link the importance of health issues related to waste management. 

The first four suggested indicators (Table 2) are related to working conditions, safety equipment and exposure to risks of the workers in urban solid waste management. Human exposure to risk factors present inn the environment is a outstanding issue in several research initiatives^10^ ^,^ ^21^. 

In the case of people working in waste management, it is essential to adopt preventive measures and using personal protective equipment^22^, needed in every phase of work to avoid the chance of accidents and aiming for safety and occupational health standards. 

In this way, the managers should include in their management plans, the conditions needed for having an integrated waste management^9^, including training courses and the use of adequate equipment to ensure safety for all persons working in this sector. 

The fifth indicator proposes the enumeration of the Dengue *foci* or proliferation of other vectors. A range of determinants foster the disease-transmitting mosquito proliferation, one of them is the inadequate disposal of waste such as bottles, tires, glasses and plastic bags, that are potential incubators for the vector^23^. In fact, in Dengue surveillance matters, inadequate waste management is considered a main factor for keeping this endemic disease going on^24^. 

Inadequate storage and disposal of waste are contributors to the environmental determinants of several diseases, being a serious public health issue in developing countries^6^. In this way, new environmentally related diseases are continually emerging and others, supposedly extinct reappear. In this study, this issue was debated when discussing the indicators six and seven, when it was proposed to note the number of cases of disease and death related to inadequate practices of waste management. 

The last indicators analyzed and proposed by the participants in the health dimension (Indicators number eight to twelve), refer to the generation and management of health services solid waste and hazardous waste. Handling this waste is a responsibility of the generator, that must have a plan for adequate management depending on the type of waste that is generated^9^, in spite of the fact that usually the municipal authority ends up performing the external handling.

A larger problem is the fact that frequently the municipal administration does not have a number of staff that are qualified and trained for performing the different phases of management in a safe way. Not withstanding this, and independently from the proposal elaborated by the institution, legal and technical principles should be observed, on the basis of an improved concern about its importance^19^.

Facilities generating hazardous waste should designate a technical officer, responsible for its waste management plan, continually revisited and yearly updated, according to the national regulations and independently of the activities of the municipal administration in direct waste management or only as auditors. Those plans should comprise the facility and activities description, definition of operational procedures for the management phases, goals and procedures for minimization of waste production, the preventive and corrective measures for its operationalization^9^.

During this process, investing in permanent training, upgrading the capacities of the workers acting directly or indirectly on the different stages of waste management, may imply an advance in the use of best practices in the waste area^10^.

The National Policy for Solid Waste states the requirements in terms of programs and actions for Environmental Education to be promoted, directed to avoid the generation, reduce, reuse and recycle solid waste. Another factor that emerges form this policy is the importance of the updated information recorded in the different sectors that are linked to waste management^9^. 

According to the participating experts in this study, the difficulties in getting hold of information and the subjectivity were a few of the characteristics seen in the indicators that were prepared. In spite of these limitations the relevance of the proposed indicators for the health dimension is key, taking into account that the knowledge gathered using these indicators may provide inputs for an integrated management directed by sustainability principles, thus minimizing impacts on the environment and reducing public health risks.

## Conclusion 

This study showed the high degree of consensus among researchers in the area of waste management that participated in this research, in relation to the sustainability indicators proposed for the health dimension. Regarding its applicability there were doubts related to the difficulties in measuring them, due to the lack of systemized data of vector proliferation and morbi-mortality related to inadequate conditions of treatment and disposal of waste. 

This issue was one of the main tenets of the activities in public health both in Brazil and Latin America, and nurses played a prominent role in health surveillance and epidemiology. Presently, in several states of Brazil, this is a key area for environmental surveillance for health.

In spite of the already mentioned setbacks, the proposed indicators may be an important guideline for updated diagnostic documents. The municipal authorities and managers at different levels of facilities, that are those that generate health services solid waste and other hazardous waste, may use these data to elaborate, roll out and evaluate in a permanent fashion their management plans. Those plans should be composed in an integrated way together with training activities for the staff members that are involved in the different sectors of the process, including the generating sources, and specially in the separating activity, that is considered one of the largest problem areas, specially concerning heath facilities. 

It is worth to remember that the sustainability indicators have a variation in the different dimensions, as they do not belong exclusively to the health area, including the economic, social, institutional and environmental areas as well. Data coming from this study are a relevant step stones for building sustainability indicators in the health area.

It is understood that the present list of indicators allows for recording and comparing data in different contexts, but in its utilization, specificities of each municipality should be considered. A next step is proposed for this research, in which the indicators may be applied in different municipalities, trying to find the potential and weaknesses of the proposed parameters. 

Not withstanding this limitation, it should be underlined the importance of the whole process of building the indicators, using as a source the suggestions of experts coming from different Brazilian regions. The suggested indicators are a relevant contribution in relation to the requirements of the NSWP and may give inputs to the policymakers and administrators in the decision-making process and follow-up the evolution of their regions, allowing additionally to examine experiences in other contexts with better indicators, looking for the development of an integrated and sustainable waste management and also to enlarge the debates on health policies.
